# Awareness and Knowledge of Pharmacists Regarding Herbal Medication and the Attitude and Demographics of their Consumers

**DOI:** 10.7759/cureus.84250

**Published:** 2025-05-16

**Authors:** Saud A Alasmari, Faisal M Al-Abdulmutaali, Faisal A Al-Rabah, Mohammad El-Nablaway

**Affiliations:** 1 College of Pharmacy, King Khalid University, Abha, SAU; 2 College of Medicine, Almaarefa University, Riyadh, SAU; 3 Department of Basic Medical Sciences, Almaarefa University, Riyadh, SAU

**Keywords:** attitudes, awareness, community pharmacists, herbal, knowledge

## Abstract

Introduction: According to the World Health Organization (WHO), herbal medicines contribute to skills and practices used in cultures worldwide to manage physical and mental disorders. In Saudi Arabia during COVID-19, herbal medicine use increased due to the perceived safety of herbal medications, especially among pregnant women and individuals with health conditions.

Objectives: The objective of this study was to determine the responsibilities and attitudes of community pharmacists toward herbal medicine use.

Methods: In this cross-sectional study, data were collected to obtain information on demographics, attitudes towards herbal medicines, safety knowledge, and the factors influencing communication. The selected sample included community pharmacists in Saudi Arabia.

Results: Among the 387 pharmacist participants, the majority were male (n=313, 80.9%) and most acquired herbal medicine knowledge by Internet research (n=146, 37.7%) and from company information (n=139, 35.9%). There was a significant association between the perceived benefit of herbal medication by pharmacists with the non-agreement of using herbal medication from unknown sources (p < 0.05), and they observed that consumers are generally unaware of their possible adverse effects. The majority of participants claimed to be effectively and frequently communicating with their patients.

Conclusion: Most participating pharmacists were male and primarily acquired herbal medicine knowledge through online resources and company information, with many recognizing both the benefits and potential risks of herbal products while actively communicating with patients. To improve public awareness and ensure safe herbal medication use, community pharmacists should receive further training and advocate for expanded research in this area.

## Introduction

The World Health Organization (WHO) defines herbal medicines as skills and practices using medicinal plants based on theories, beliefs, and experiences unique to many cultures, used to prevent, diagnose, improve, or treat physical and mental disorders, forming a comprehensive body of knowledge [1]. Herbal medicines are increasingly used for the management of diseases due to a widely-held belief in their low toxicity [2]. Herbal medicine is extensively utilized in the Arab world because of the perception of inherent safety. In Saudi Arabia, herbal medicine is widely used by individuals with various health conditions, particularly among pregnant women [3]. Overall, women tend to have a more positive attitude towards herbal medicines and are more likely to use them compared to men [4]. Older healthcare professionals have more experience and are more aware of the possible safety issues of herbal medications [5]. Most consumers take herbal medications without a prescription because they believe these medicines are safe and effective [6], influenced by cultural beliefs and occasional positive outcomes, reducing reliance on conventional drugs [7].

The pharmacist plays an important role in educating patients on herbal products, whether medicines or food supplements [8]. In the Middle East, people acknowledge the importance of the pharmacist’s role [9]. Pharmacists in Saudi Arabia possess good knowledge of the safety and efficacy of herbal medications, enabling them to mitigate the unnecessary use [10]. Previous studies in Saudi Arabia have highlighted varying levels of pharmacists' knowledge and attitudes toward herbal medicines. Many pharmacists believe in the safety and effectiveness of these products and support the idea that herbal medicines should be dispensed through pharmacies to ensure proper guidance [11]. Despite this awareness, pharmacists often encounter challenges in practice, particularly in verifying the purity and quality of herbal products, as sensory evaluation and official certification are frequently unavailable. Barriers such as time constraints, lack of reliable resources, and incomplete scientific evidence further limit pharmacists' ability to counsel patients effectively on herbal product safety [12]. Additionally, public reliance on herbal information from non-professional sources such as social media, family, and friends is widespread, which may contribute to misinformation and unsafe practices [13]. There are many contributing factors affecting drug safety, and pharmacists rarely disclose the indications, side effects, and interactions of herbal medicines [14]. The barriers that pharmacists face in herbal counseling are the lack of time, reliable sources, scientific evidence supporting the use of herbal medicines, knowledge, and interest. Indeed, pharmacists must initiate the discussion more than patients [15]. A systematic review and meta-analysis showed that the harmful effects of commonly consumed herbal medicines during pregnancy are not fully understood, making it difficult to rule out teratogenic effects [16]. The majority of pregnant women indicated that they would consider using herbal medicines during pregnancy if advised by a healthcare professional [17].

Herbal products can potentially have toxicity and cause harm to consumers [18]. To ensure optimal therapeutic outcomes, pharmacy practitioners must be aware of herbs and their interactions with drugs, as they influence drug pharmacokinetics and pharmacodynamics [19]. Recent studies have shown positive effects in curing diseases and substantial improvement in patients’ symptoms and overall quality of life [20-24]. Healthcare professionals are mostly familiar with drug-herbal interactions (83%), but further research is needed to identify the awareness of pharmacists toward these interactions [25]. During the COVID-19 pandemic, the utilization rate of herbal medication and food supplements in Saudi Arabia was high [26], and pharmacists were responsible for advising and dispensing herbal medicines to patients in pharmacies.

Despite the growing reliance on herbal treatments, there are gaps in pharmacists' knowledge about herb-drug interactions, potential side effects, and effective communication with patients. Our objectives in this study are, thus, to determine the knowledge of community pharmacists regarding herbal medication, pharmacist beliefs regarding herbs, the knowledge of pharmacists on herbal indications and their herb-drug interactions, awareness of the patient of possible adverse effects of herbal medication, and the factors that contribute to communication between the pharmacist and their patient. This study is necessary to explore pharmacists' understanding and beliefs regarding herbal medicines, their awareness of adverse effects, and pharmacist-patient communication, with the aim of improving the safe and effective use of herbal products in community pharmacy settings.

## Materials and methods

This was a cross-sectional study with a self-administered questionnaire for community pharmacists in Saudi Arabia, conducted between July and September 2023. The study was approved by the Institutional Review Board of AlMaarefa University (approval number: IRB23-023). All participants had the right to participate or withdraw by filling out the informed consent form before beginning the questionnaire, and the study complied with the tenets of the Declaration of Helsinki.

Participants

According to the Ministry of Health in Saudi Arabia, there are 34,040 licensed pharmacists practicing in the country [27]. Community pharmacists practicing in Saudi Arabia with a diploma, bachelor’s degree, Pharm. D. degree, master’s degree, or PhD were considered eligible for the study. Inclusion criteria were pharmacists licensed in Saudi Arabia who were willing to participate. Pharmacists unwilling to participate were excluded. 

Sample size and sampling procedure

The expected sample size of around 380 participants was estimated using a Raosoft sample size calculator (www.raosoft.com, Raosoft Inc., Seattle, Washington, United States) with a confidence level of 95% and a margin of error of 5%. The sampling procedure was a non-probability convenience method. 

The survey was distributed via Google Forms (Google LLC, Mountain View, California, United States) in collaboration with Nahdi Medical Company, Jeddah, Saudi Arabia) and through visits to 237 additional community pharmacies across the country. Of the 411 community pharmacists contacted, 387 (94.2%) responded, while 24 (5.8%) declined to participate.

Study tool and data collection

The questionnaire used in the study is given in the Appendices. A pilot test was conducted to evaluate the validity and reliability of the questionnaire. All community pharmacists successfully completed the questionnaire during the pilot test. Reliability was assessed using Cronbach's α, which demonstrated acceptable internal consistency with a value of 0.732. To minimize selection bias, the survey was distributed in collaboration with Nahdi Medical Company to ensure wide coverage among their pharmacists. Additionally, multiple visits were made to various pharmacies to confirm that only pharmacists completed the survey. The questionnaire, designed and distributed using Google Forms, was structured by the authors and consisted of two sections: Demographic and background information: Included gender, age, marital status, location, highest qualification, professional level, contract status, work schedule, and years of experience; Pharmacist attitudes toward patients’ consumption of herbal medicines: Comprised 29 questions assessing knowledge and awareness of herbal medicines, factors influencing pharmacist-patient communication, beliefs about herbal medicines, and perspectives on patient awareness of adverse effects and herb-drug interactions. The informed consent form clarified that participant data was used only for research purposes.

Statistical analysis

Data was collected and cleaned in an Excel sheet (Microsoft Corporation, Redmond, Washington, United States) and then entered for analysis using the IBM SPSS Statistics for Windows, Version 26.0 (Released 2019; IBM Corp., New York, United States). Data underwent a non-parametric test and descriptive statistics analysis, including frequencies and percentages, to summarize results. To assess the association between independent variables, the chi-square test was used for identifying the relationship between consumers' age and sex, the benefit of using herbal products procured from a pharmacy or other sources, satisfaction regarding quality and price of herbal medications, and any language barrier that may compromise communication. Spearman’s rank correlation coefficient was used to assess the relationship between pharmacists' awareness of herbal adverse effects and the incidence of allergic reactions, as well as the association between language barriers and communication challenges. P-value < 0.05 was considered significant.

**Figure 1:**
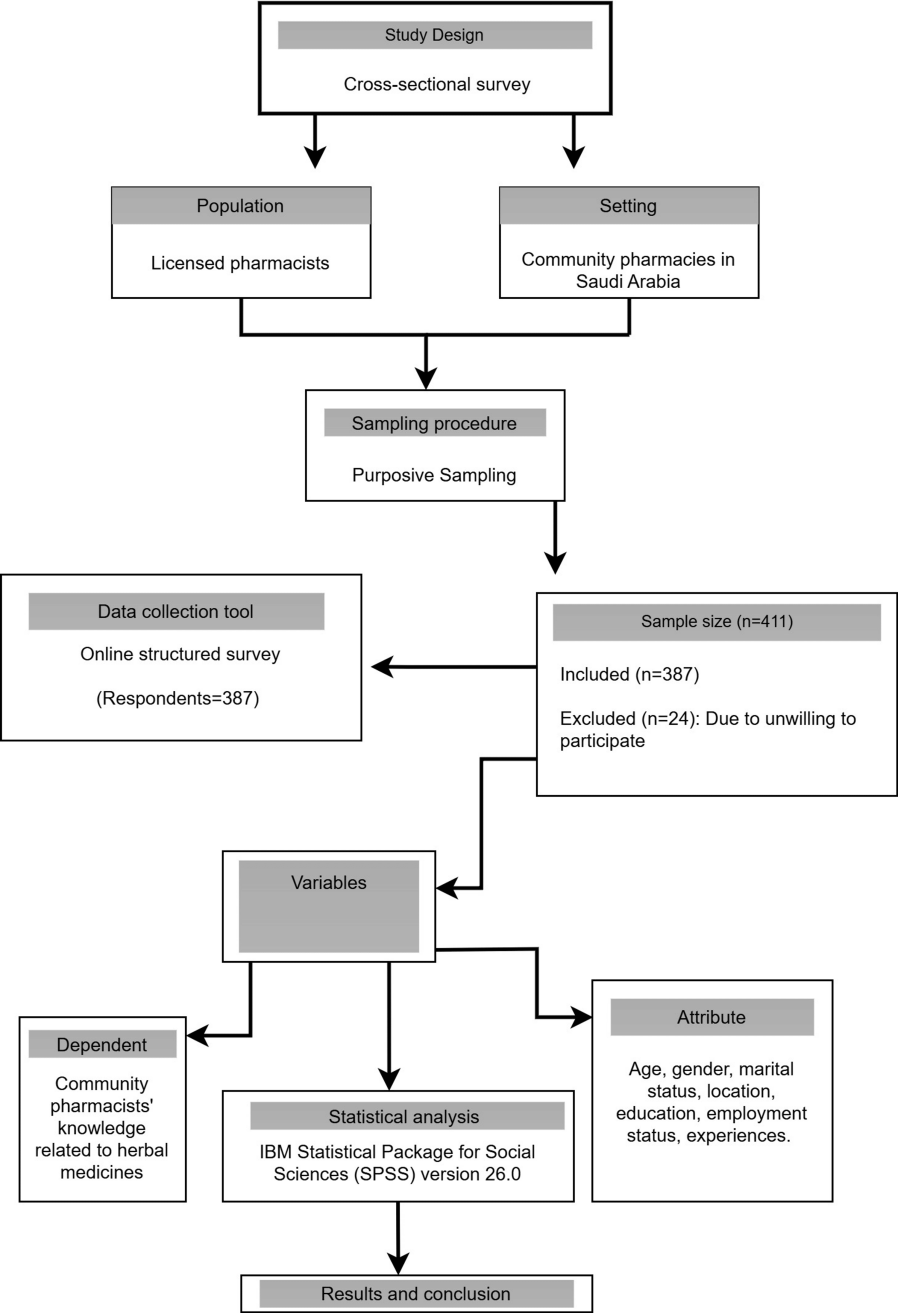
Outline of research design and procedure.

Details of the research design and procedure are in Figure 1. 

## Results

Out of 411 participants, 387 (94.2%) pharmacists completed the questionnaire. Among the 387 who completed the questionnaire, 313 (80.9%) participants were male and 74 (19.1%) were female. The average age of participants was between 26 and 35 years. The participating pharmacists were qualified with a bachelor’s degree (n=230, 59.4%), Pharm. D. degree (n=132, 34.1%), master’s degree (n=19, 4.9%), diploma (n=5, 1.3%), and PhD (n=1, 0.3%). Most pharmacists had more than two years’ experience in community pharmacies (n=307, 79.3%), and 51 (13.2%) had attended special courses in alternative medicine. Almost all the pharmacists worked in cities (n=360, 93.0%). In terms of employment, 361 (93.3%) participants worked full-time jobs (Table 1).

**Table 1:** Characteristics of the participating community pharmacists (N=387)

Variables	Frequency	Percentage
Sex		
Male	313	80.9%
Female	74	19.1%
Age groups (years)		
20-25	44	11.4%
26-30	146	37.7%
31-35	118	30.5%
More than 35	79	20.4%
Marital Status		
Single	142	36.7%
Married	240	62.0%
Divorced	1	0.3%
Widow/Widower	4	1.0 %
Work location		
City	360	93.0%
Village	27	7.0%
Highest qualification		
Diploma	5	1.3%
Bachelor’s degree	230	59.4%
Pharm D	132	34.1%
Master's degree	19	4.9%
PhD	1	0.3%
Professional level		
Technician	7	1.8%
Pharmacist	341	88.1%
Senior pharmacist	34	8.8%
Consultant Pharmacist	5	1.3%
Contract status:		
Full time	361	93.3%
Part-time	24	6.2%
Temporary/Casual	2	0.5%
Shifts		
Yes	275	71.1%
No	112	28.9%
Experience		
Less than 2 years	80	20.7%
2 to 5 years	105	27.1%
6 to 10 years	100	25.8%
More than 10 years	102	2S
Special courses in alternative medicine		
Yes	51	13.2%
No	336	86.8%

In terms of acquiring information, 146 (37.7%) pharmacists relied on Internet research, 139 (35.9%) obtained information from their company, 79 (19.6%) relied on their experience, and 26 (6.7%) had other sources of information (Table 2). According to the majority of pharmacists (n=225, 58.1%), most consumers requested herbal medications with a prescription, while 162 (41.9%) reported most customers did not present prescriptions. Most pharmacists (n=371, 95.9%) mentioned the need for more studies on herbal medication usage. Concerning the reasons for pharmacists not to dispense herbal medication, the majority (n=121; 31.3%) reported that patients do not request herbal products while 105 (27.1%) pharmacists reported that it was because they identified a drug interaction with their use, 46 (11.9%) believed there was no benefit, and 115 (29.7%) had other reasons. Furthermore, a large number of pharmacists (n=215, 55.6%) sometimes observed wrong beliefs among herbal medication consumers, while 48 (12.4%) rarely did. Most participants (n=190, 49.1%) attended between 100 and 200 patients daily, 141 (36.3%) attended less than 100, and 56 (14.5%) attended more than 200. Regarding direct communication with their patients, most pharmacists (n=156, 40.3%) stated this is usually the situation and 147 (38.0%) stated this happened always.

**Table 2:** Experience and knowledge of pharmacists and their responses about customer demographics, herbal medicine use, and knowledge

Variables	Frequency	Percentage
Patients/customers served daily		
Less than 100	141	36.3%
100 – 200	190	49.1%
More than 200	56	14.5%
Direct communication with patients/customers		
Never	6	1.6%
Rarely	10	2.6%
Sometimes	68	17.6%
Usually	156	40.3%
Always	147	38.0%
Sources for information about herbal medication		
Company-provided sources	139	35.9%
Research on the Internet	146	37.7%
Own experience	76	19.6%
Other sources	26	6.7%
From your observation of patients/customers, who mostly use herbal medications?		
Male	190	49.1%
Female	197	50.9%
From your observation of patients/customers, what is the average age of the users of herbal medications?		
Less than 20	4	1.0%
20 – 25	11	2.8%
26 – 30	32	8.3%
More than 30	340	87.9%
Encounter prescriptions for herbal medications?		
Yes	225	58.1%
No	162	41.9%
Do you agree with using herbal medications from unknown sources?		
Strongly disagree	292	75.5%
Disagree	56	14.5%
Neither agree nor disagree	36	9.3%
Agree	2	0.5%
Strongly agree	1	0.3%
Do you agree with using homemade herbal mixtures?		
Strongly disagree	118	30.5%
Disagree	101	26.1%
Neither agree nor disagree	138	35.7%
Agree	27	7.0%
Strongly agree	3	0.8%
Satisfaction with the quality of herbal medication products in community pharmacies		
Very dissatisfied	11	2.8%
Dissatisfied	12	3.4%
Neither dissatisfied nor satisfied	135	34.9%
Satified	186	48.1%
Very satisfied	42	10.9%
Satisfaction of herbal medication users with prices in community pharmacy		
Very dissatisfied	29	7.5%
Dissatisfied	96	24.8%
Neither dissatisfied nor satisfied	186	48.1%
Satisfied	71	18.3%
Very satisfied	5	1.3%
Need for more research/studies about herbal medications		
Yes	371	95.9%
No	16	4.1%
Have you ever encountered a pregnant woman requesting herbal medications that could potentially harm the fetus?		
Yes	116	30.0%
No	271	70.0%
Any drug interactions with the use of herbal medications in patients/customers		
Yes	16	4.1%
No	371	95.9%
If you were to encounter an interaction, what type would it most likely be?		
Herbal-Drug interaction	135	34.9%
Herbal-Disease interaction	97	25.1%
Herbal-Herbal interaction	7	1.8%
Others	148	38.2%
Do you see patients/customers having allergies from using some herbal medications?		
Never	116	30.0%
Rarely	155	40.1%
Sometimes	98	25.3%
Usually	12	3.1%
Always	6	1.6%
Awareness of herbal medication users about its adverse effects associated with herbal use		
Very unaware	39	10.1%
Unaware	154	39.8%
Neither aware nor unaware	156	40.3%
Aware	33	8.5%
Very aware	5	1.3%
Reasons for not dispensing herbal medications to patients/customers		
There is interaction in their use	105	27.1%
There is no benefit in their use	46	11.9%
No demand by patients/customers	121	31.3%
Others	115	29.7%
Observation of wrong beliefs among herbal medication users		
Never	24	6.2%
Rarely	48	12.4%
Sometimes	215	55.6%
Usually	74	19.1%
Always	26	6.7%
In your opinion, do you think that some herbal medications may worsen a patient's condition?		
Strongly disagree	11	2.8%
Disagree	50	12.9%
Neither agree nor disagree	144	37.2%
Agree	137	35.4%
Strongly agree	45	11.6%

Table 3 shows that the majority of the participating pharmacists reported customers to be primarily in the age group of > 30 years, of which 165 reported as primarily female and 175 reported as primarily male. As per their observations, there were significant age-related differences among their patients, with females constituting the majority (n=197, 50.9%) (p < 0.05).

**Table 3:** Association between age group of consumers with sex as reported by the study participants Χ2: Chi-square test. *p<0.05 *considered significant.

Age (years)	Sex (n)		P-value	Χ2 test score
	Male	Female		
< 20 years (n=4)	3	1	0.003	9.9
20 – 25 years (n=11)	4	7		
26 – 30 years (n=32)	8	24		
> 30 years (n=340)	175	165		

Regarding pharmacists’ opinions on herbal medications, the majority of the pharmacists strongly disagreed with using herbal medications procured outside pharmacies and believed there are benefits to using herbal products from pharmacies for certain symptoms (Table 2). Table 4 shows the association between perceived benefits and agreement with using herbal medication from unknown sources (p < 0.05).

**Table 4:** Association between perceived benefits of herbal medicines and acceptance of unknown sources Χ2: Chi-square test. p<0.05 considered significant.

Perceived benefit of herbal medication	Agreement with using herbal medications from unknown sources (n)					P-value	Χ2 test score
	Strongly disagree	Disagree	Neutral	Agree	Strongly agree		
Very poor (n=12)	9	0	3	0	0	0.005	49.2
Poor (n=11)	3	4	3	1	0		
Acceptable (n=115)	86	12	17	0	0		
Good (n=188)	144	33	10	1	0		
Very good (61)	50	7	3	0	1		

In terms of satisfaction with the quality of herbal medications, 186 (48.1%) pharmacists were satisfied, 96 (34.9%) were neither satisfied nor dissatisfied, 42 (10.9%) were very satisfied, 12 (3.4%) were dissatisfied, and 11 (2.8%) were very dissatisfied (Table 2). Additionally, most pharmacists were neutral regarding the consumers' satisfaction with the prices of herbal medicines in community pharmacies. Table 5 presents the association between participants' satisfaction with the quality and the consumers' satisfaction with the pricing.

**Table 5:** Participants satisfaction with quality and consumers' percieved satisfaction with pricing Χ2: Chi-square test. p<0.05 considered significant

Pharmacists' satisfaction with quality	Consumers' percieved satisfaction with price (n)					P-value	Χ2 test score
	Very dissatisfied	Dissatisfied	Neutral	Satisfied	Very satisfied		
Very dissatisfied (n=11)	7	2	2	0	0	< 0.001	93.7
Dissatisfied (n=13)	3	4	4	2	0		
Neutral (n=135)	10	32	82	11	0		
Satisfied (n=186)	8	50	79	47	2		
Very satisfied (n=42)	1	8	19	11	3		

Most pharmacists (n=271, 70.0%) reported that they did not encounter requests from pregnant women for herbal medication that may harm the fetus. Most pharmacists (n=155, 40.1%) rarely saw patients having allergies from using herbal medications, and they believed that consumers were mostly either unaware (n=154, 39.8%) or neither aware nor unaware (n=156, 40.3%) of adverse effects associated with the use of herbal medications (Table 2). Table 6 shows the association between awareness of adverse reactions and consumers having allergies.

**Table 6:** Association between the reported awareness among consumers regarding adverse reactions and encounters with consumers having allergies ρ: Spearman’s rho test. p<0.05 considered significant.

Adverse reactions awareness	Consumers having allergy (n)					P-value	ρ test coefficient
	Never	Rarely	Sometimes	Usually	Always		
Very unaware (n=39)	14	15	6	3	1	0.148	0.074
Unaware (n=154)	43	74	32	4	1		
Neutral (n=156)	49	54	47	4	2		
Aware (n=33)	7	12	12	1	1		
Very aware (n=5)	3	0	1	0	1		

Table 7 shows the ease of communication of the pharmacists with their customers/patients and their opinions regarding the attitude of customers/patients about herbal medicine. In terms of difficulty in understanding the accent of patients, 184 (47.5%) participants stated that this occurred rarely, while only five (1.3%) faced this usually. Concerning consumers accepting advice from pharmacists, most (n=152, 39.3%) believed their patients sometimes accepted their advice, while 140 (36.2%) felt their advice was usually accepted. In terms of reading the scientific names of herbal medications, 148 (38.2%) pharmacists sometimes had difficulties, while 122 (31.5%) rarely did. The main causes of requests for herbal medications from patients are personal beliefs (n=268, 69.3%), prescriptions (n=61, 15.8%), and others (n=58, 15.0%). Participants stated that the purposes of the requests were for treatment (n=237, 61.2%), for prevention (n=76, 19.6%), for cosmetics (n=67, 17.3%), and seven (1.8%) reported other purposes. Regarding familiarity with using herbal medication, 182 47.0%) participants were somewhat familiar, 129 (33.3%) were familiar, 35 (9.0%) were unfamiliar, 25 (6.5%) were very unfamiliar, and 16 (4.1%) were very familiar. Moreover, most participants (n=155, 40.1%) sometimes preferred herbal medications over conventional medicines, while 26 (6.7%) always did.

**Table 7:** Pharmacist's ease of communication and attitudes toward patients' consumption of herbal medicines

Variables		Frequency	Percentage	
Any difficulties understanding patients/customers language?				
Never	125			32.3 %
Rarely	194			50.1 %
Sometimes	64			16.5 %
Usually	3			0.8 %
Always	1			0.3 %
Any difficulties understanding patients/customers accent?				
Never	115			29.7 %
Rarely	184			47.5 %
Sometimes	73			18.9 %
Usually	5			1.3 %
Always	10			2.6 %
Main causes of taking herbal medications by patients/customers				
Prescription	61			15.8 %
Personal beliefs	268			69.3 %
Others	58			15.0 %
From your opinion how widespread is the culture of herbal medication use?				
Very unfamiliar	25			6.5 %
Unfamiliar	35			9.0 %
Somewhat familiar	182			47.0 %
Familiar	129			33.3 %
Very familiar	16			4.1 %
Most important reason for the demand for herbal medications				
For therapeutic purpose	237			61.2 %
For cosmetic purpose	67			17.3 %
For preventive purpose	76			19.6 %
Others	7			1.8 %
Any difficulties reading the scientific names of herbal medications				
Never	89			23.0 %
Rarely	122			31.5 %
Sometimes	148			38.2 %
Usually	22			5.7 %
Always	6			1.6 %
Clear benefit for using herbal medication in certain symptoms like cough, headache, constipation, etc.				
Very poor	12			3.1 %
Poor	11			2.8 %
Acceptable	115			29.7 %
Good	188			48.6 %
Very good	61			15.8 %
Do you prefer starting the patient on herbal medications rather than pharmaceutical drugs?				
Never	34			8.8 %
Rarely	87			22.5 %
Sometimes	155			40.1 %
Usually	85			22.0 %
Always	26			6.7 %
Acceptance from patients/customers to your advice/guide about using herbal medications				
Never	12			3.1 %
Rarely	43			11.1 %
Sometimes	152			39.3 %
Usually	140			36.2 %
Always	40			10.3 %
Have you succeeded in changing the wrong beliefs of herbal medication users?				
Never	24			6.2 %
Rarely	49			12.7 %
Sometimes	204			52.7 %
Usually	89			23.0 %
Always	21			5.4 %

Table 8 presents the association between understanding the language of consumers and the advice taken by the consumers. Most participants rarely encountered difficulties in understanding the language. However, a significant challenge was associated with changing patients' wrong beliefs (p < 0.05). 

**Table 8:** Association between pharmacists' ease of communication with their success in changing wrong beliefs in herbal medicine consumers ρ: Spearman’s rho test. p<0.05 considered significant.

Understanding language	Success in changing the wrong beliefs of herbal medication users (n)					P-value	ρ test coefficient
	Never	Rarely	Sometimes	Usually	Always		
Never (n=125)	9	13	62	26	15	0.06	-0.096
Rarely (n=194)	11	26	100	55	2		
Sometimes (n=64)	3	10	39	8	4		
Usually (n=3)	1	0	2	0	0		
Always (n=1)	0	0	1	0	0		

## Discussion

Our study aimed to clarify community pharmacists' beliefs and challenges regarding herbal medicines in Saudi Arabia. The findings reveal that pharmacists in the region attend to a high volume of patients daily, positioning them as key players in advising on herbal product use. This aligns with previous research emphasizing the critical role of pharmacists in ensuring the safe dispensing of herbal medicines [8]. However, while our study found that 37.7% of Saudi pharmacists rely on online sources for herbal medicine information, a study in Riyadh reported a higher dependence on textbooks (54%) and lower internet usage (19%) [12]. This discrepancy may reflect evolving trends in information-seeking behavior, possibly due to increased digital accessibility. Such comparisons highlight the need for standardized, up-to-date educational resources to ensure consistent knowledge dissemination among pharmacists.

The rising demand for herbal medicines in community pharmacies raises important questions about contributing factors and associated challenges. Our data indicate that women (53.6%) and older individuals are more likely to use herbal products, consistent with global trends showing higher herbal medicine use among women and older adults. Interestingly, while Saudi pharmacists actively engage in herbal product dispensing, studies in the United States reveal reluctance due to insufficient knowledge about safety and efficacy [14]. This contrast underscores regional differences in pharmacists' confidence and training regarding herbal medicines, suggesting a need for targeted educational interventions in regions where herbal medicine literacy is lacking.

A key finding of the current study is pharmacists' distrust of herbal products obtained from non-pharmacy sources, reflecting concerns about quality and safety. This aligns with research documenting risks associated with unregulated herbal products, including contamination and adulteration [28]. However, the participants of the current study reported no observed cases of herbal-drug interactions, contrasting with literature highlighting such risks [29]. This difference may stem from underreporting or lack of awareness, emphasizing the need for improved pharmacovigilance systems to detect and document herbal-related adverse events.

Regarding pregnancy, the findings of the current study suggest that most pregnant women in Saudi Arabia are cautious about herbal use, consistent with a study advocating for better education on herbal risks during pregnancy [17]. However, a study found that most patients with chronic diseases were unaware of the risks associated with herbal supplements, with many not consulting or informing healthcare providers [30]. This comparison highlights the importance of culturally tailored patient education to address knowledge gaps.

Pharmacists in the current study demonstrated strong communication skills, effectively addressing patients' personal beliefs about herbal medicines. As shown in Table 8, when language barriers are absent, patients' misconceptions are eliminated. This aligns with research showing that pharmacist-patient interactions significantly influence herbal medicine use [31]. However, patient adherence to professional advice can vary depending on cultural and personal health beliefs, as seen in studies on medication compliance [32]. These findings suggest that pharmacist training should incorporate strategies for navigating patient perspectives while promoting evidence-based use of herbal products.

While the current study provides valuable insights, several limitations must be acknowledged. The three-month survey period had limited participation, and the underrepresentation of rural pharmacists could affect generalizability. Additionally, reliance on self-reported data introduces potential response bias. Future studies should extend data collection periods, include more rural participants, and incorporate observational methods to validate findings. Despite these limitations, our study contributes to understanding pharmacists' perspectives on herbal medicines in Saudi Arabia. By comparing our results with global literature, we highlight regional variations and universal challenges, underscoring the need for standardized guidelines and continuous professional development in herbal medicine safety.

## Conclusions

This study provides valuable insights into the extent of knowledge, as well as the perspectives and attitudes of community pharmacists and their patients regarding herbal medicine in Saudi Arabia. Pharmacists have many responsibilities, including educating the patient, ensuring the safety of herbal medicine use, and keeping up to date with the latest medicinal herbal product research. The main challenges faced by the community pharmacist are limited time and access to reliable sources of information. Despite this, pharmacists have proven their ability to provide better quality care to patients requesting herbal products. Overall, our findings contribute to promoting pharmacists’ views on herbal medicines and highlight the need for more research to improve the safety of herbal medication use.

## Appendices

Questionnaire

**Table 9:** Section 1: Demographic and background information

Questions	
What is your gender?	
Male	
Female	
How old are you?	
What is your marital Status?	
Single	
Married	
Divorced	
Widowed	
Where is your work located?	
City	
Village	
What is your highest qualification?	
Diploma	
Bachelor’s	
Pharm D	
Master’s	
PhD	
According to the Saudi Commission for Health Specialties, which category describes your professional level?	
Technician	
Pharmacist	
Senior pharmacist	
Consultant Pharmacist	
What is your employment contract status?	
Full time	
Part-time	
Temporary/Casual	
Do you work in shifts?	
Yes	
No	
Do you have any experience in community pharmacy, for how many years?	
Less than 2 years	
2 to 5 years	
6 to 10 years	
More than 10 years	
Do you have any Diploma or special courses in alternative medicine or herbal medications?	
Yes	
No	

**Table 10:** Section 2: Pharmacist attitudes toward patients' consumption of herbal medicines

How many patients/customers do you serve daily?		
Less than 100		
100 – 200		
More than 200		
How frequently do you directly interact with patients/Customers?		
Never		
Rarely		
Sometimes		
Usually		
Always		
What are your sources that you used for herbal medication?		
Company-provided sources		
Research in internet		
Depend on my experience		
Others		
From your observation to patients/customers, who uses herbal medications?		
Male		
Female		
From your observation to patients/customers, what is the average age of the users for herbal medications?		
Less than 20		
20 – 25		
26 – 30		
More than 30		
Through your practice, do you encounter prescriptions containing any herbal medications?		
Yes		
No		
Do you agree with using herbal medications from unknown sources?		
Strongly disagree		
Disagree		
Neither agree nor disagree		
Agree		
Strongly Agree		
Do you agree with using homemade herbal mixtures?		
Strongly disagree		
Disagree		
Neither agree not disagree		
Agree		
Strongly agree		
Are you satisfied with the quality of herbal medications products in community pharmacies?		
Very dissatisfied		
Dissatisfied		
Neither dissatisfied nor satisfied		
Satified		
Very satisfied		
How satisfied are herbal medication users with prices in community pharmacy?		
Very dissatisfied		
Dissatisfied		
Neither dissatisfied nor satisfied		
Satified		
Very satisfied		
Do you think that society needs more research/studies about herbal medications?		
Yes		
No		
Have you ever seen a pregnant woman want to dispense any herbal medications that may harm the fetus?		
Yes		
No		
Did you find during your work period any interaction with uses of herbal medications to patients/customers		
Yes		
No		
If there is any interaction, what is the type of interaction?		
Herbal-Drug interaction		
Herbal-Disease interaction		
Herbal-Herbal interaction		
Others		
Do you see patients/Customers having allergies from using some herbal medications?		
Never		
Rarely		
Sometimes		
Usually		
Always		
How do you see the awareness of herbal medications users about its adverse effects associated with herbal use?		
Very unaware		
Unaware		
Neither aware not unaware		
Aware		
Very aware		
What are the reasons that you do not dispense herbal medications for patients/customers		
There is interaction in their use		
There is no benefit in their use		
Not demand by patients/Customers		
Others		
Have ever you observed wrong beliefs among herbal medications users?		
Never		
Rarely		
Sometimes		
Usually		
Always		
From your opinion, do you think that some herbal medications may cause a patient's condition worse?		
Strongly disagree		
Disagree		
Neither agree nor disagree		
Agree		
Strongly agree		
Any difficulties understanding patients/customers language?		
Never		
Rarely		
Sometimes		
Usually		
Always		
Any difficulties understanding patients/customers accent?		
Never		
Rarely		
Sometimes		
Usually		
Always		
Main causes of taking herbal medications by patients/customers		
Prescription		
Personal beliefs		
Others		
From your opinion how widespread is the culture of herbal medication use?		
Very unfamiliar		
Unfamiliar		
Somewhat familiar		
Familiar		
Very familiar		
Most important reason for the demand for herbal medications		
For therapeutic purpose		
For cosmetic purpose		
For preventive purpose		
Others		
Any difficulties reading the scientific names of herbal medications		
Never		
Rarely		
Sometimes		
Usually		
Always		
Clear benefit for using herbal medication in certain symptoms like cough, headache, constipation, etc.		
Very poor		
Poor		
Acceptable		
Good		
Very good		
Do you prefer starting the patient on herbal medications rather than pharmaceutical drugs?		
Never		
Rarely		
Sometimes		
Usually		
Always		
Acceptance from patients/customers to your advice/guide about using herbal medications		
Never		
Rarely		
Sometimes		
Usually		
Always		
Have you succeeded in changing the wrong beliefs of herbal medication users?		
Never		
Rarely		
Sometimes		
Usually		
Always		
